# Cancer incidence in Morocco: report from Casablanca registry 2005-2007

**DOI:** 10.11604/pamj.2013.16.31.2791

**Published:** 2013-09-29

**Authors:** Zineb Bouchbika, Houssam Haddad, Nadia Benchakroun, Houda Eddakaoui, Souad Kotbi, Anis Megrini, Hanane Bourezgui, Souha Sahraoui, Marilys Corbex, Mhamed Harif, Abdellatif Benider

**Affiliations:** 1 Department of Radiotherapy-oncology, Ibn Rochd University Hospital, Casablanca, Hassan II University, Morocco; 2 Registry team, Regional Directorate of Health of Casablanca, Morocco; 3 Department of Public Health, Institute of Tropical Medicine, Antwerp, Belgium; 4 Centre Hospitalier Mohammed VI, Marrakech, Morocco

**Keywords:** Population-based cancer registry, cancer incidence, Casablanca, Morocco

## Abstract

**Introduction:**

Few population-based cancer registries are in place in developing countries. In order to know the burden of cancer in Moroccan population, cancer registry initiative was put in place in the Casablanca district, the biggest city of Morocco.

**Methods:**

The data collected covers 3.6 millions inhabitant and included Casablanca city and the administrative region.

**Results:**

The data collected in the years 2005-07 show that the top 5 forms of cancers in women were breast (ASR: 36.4 per 100,000), cervical (15.0), thyroid (6.7), colon-rectum (5.8), and ovarian (5.3); the top 5 cancers in men were lung (25.9), prostate (13.5), bladder (8.7), colon-rectum (8.1) and non-Hodgkin lymphoma (7.2). Tumours of haematopoietic and lymphoid tissues represented 11% of all cancers (skin excluded); some presented unusual sex ratios. For breast, cervical, colorectal and thyroid cancer, respectively 57%, 42%, 28% and 60% of the cases were under 50 years of age. This was attributable to particularly low numbers of cases recorded among old people, and the young age of the general population; the observed age-specific incidences under age 50 were not higher than in western countries. Cancers at young ages were particularly common in women: 67% of the cases were under 50. Stage at diagnosis could be obtained for 82% of the breast cancer cases and was as follows: 28% local, 63% regional and 9% distant, in the absence of screening.

**Conclusion:**

These first population-based data have provided an invaluable resource for the national cancer control plan of Morocco, and will be useful tool to its future evaluation.

## Introduction

Differences in cancer incidence among different populations reflect the impact of environmental and genetic factors in cancer occurrence [1]. In Morocco, only hospital based registries are available. The main cancer centres are based in Casablanca and Rabat. The cancer centre of Casablanca is set up since 1929 and the centre of Rabat in 1985. According to the archives of the Ibn Rochd oncology centre in Casablanca, cervical cancer was almost the only form of cancer in women in Morocco in the 1940s [2]. It remained the most frequent cancer in the population until the 1990s, when a significant increase of breast cancer in women and lung cancer in men were observed [2]. These same observations were reported by the National Institute of Oncology in Rabat [2].

In 2005, the Foundation Lalla Salma for prevention and treatment of cancer was created by Princess Lalla Salma, and its main objective was the establishment of a national plan to prevent and control cancer in Morocco. The absence of incidence data was an obstacle. The Foundation Lalla Salma supported the pre-existing initiative of a group of university teachers of the Faculty of Medicine and Pharmacy who wanted to set up a population-based registry covering the Greater Casablanca district. The data of 3 first years of activity of the cancer registry are presented in this article.

## Methods

In 2006 and 2007 numerous meetings were held by the group of university teachers to raise awareness about the registry mission and the requirements among all the doctors of the Greater Casablanca district (generalists and specialists from the public and private sector), and notably among the 17 pathology laboratories of the district. In 2007, the first report from the Greater Casablanca population-based registry was published (in French): it provided incidence of all types of cancer in the district for the year of 2004 [3]. A second report has just been published that covers 3 consecutive years from 2005 to 2007, and in which stage at diagnosis for the 13 most common cancers has been actively sought [4]. The data corresponding to this last report are presented and discussed here.

### Population

The Greater Casablanca district is the most populated area of Morocco and hosts 12% of the total population of the country. It spans over 1115 km^2^ and counts 3 615 903 inhabitants, rather more women (1 833 648) than men (1 782 255); 63% of the population is under 35 years of age. National population censuses are performed in Morocco every 10 years, the last one took place in 2004. The Haut Commissariat au Plan (National Institute of Statistics) provides estimates of the growth rate of the Moroccan population by each age-sex group and by year since 2004 [5]. These rates were used to prepare estimates of the Greater Casablanca population in the period 2005 - 2007 [4].

The population of the Greater Casablanca district is mainly urban (91.6% vs. 8.4% rural) and is concentrated in the city of Casablanca. The population of Morocco as a whole is less urban than the Greater Casablanca district (55% vs. 45% rural) [6]. However, being the economic centre of the country, Casablanca attracts a lot of migrants from all regions of Morocco, including the rural regions and these results in a very large socio-economic heterogeneity. The Greater Casablanca population can, therefore, be considered as reasonably representative of the Moroccan population.

### Data collection and cancer classification

The registry is located in the University Hospital of Casablanca (UHC), which is the only public facility that provides radiotherapy in the region. The collection of cases for years 2005-2007 began in 2008 and was performed in an active way. During 2 years, 3 registrars, assisted by 10 medical students and 2 MDs from the public health institute ("Observatoire de la Santé Publique") visited the relevant health institutions of the region and filled in registry forms for all medical records/pathology reports mentioning cancer. The relevant institutions included 17 pathology laboratories (2 public ones and 15 private ones), 11 public hospitals (including the UHC) and 10 private hospitals. Data from death certificates were not used as in 90% of the cases the real cause of death was not mentioned.

Information recorded on each case included name, date of birth or age, sex, residence, incident date, hospital, basis of diagnosis, tumour site and histology, stage of disease, TNM, extension (local, regional, distant) and vital status. Tumour site and histology were coded using the International Classification of Diseases for Oncology, Third edition (ICD-O-3). For tabulation of results, these were converted to the 10th revision of the ICD [7]. Data were entered, standardized and processed using Epi-info and Excel softwares; they were examined to ensure that only incident cases were recorded and that multiple reports on the same individual were counted only once. Cases with an unknown address were excluded. Confidentiality aspects were handled carefully and a confidentiality charter was signed by every team member.

### Statistical analyses

Crude incidence rate (CR) and age-specific incidence rate (ASR) were calculated as described in the IARC Cancer in 5-continent-publications [8]. Age standardisation was performed by the direct method [9]. The 95% confidence intervals (CIs) of ASRs were calculated according to the classical method [10].

## Results

### Quality of data

In total 25,773 cases were registered for the three years 2005-2007. Among them 13,115 (50.9%) were duplicates and 735 cases (2.8%) were excluded due to the absence of data about their address. A total of 11,923 cancer cases were retained, of which 5,551 (46.6%) were men and 6,372 (53.4%) women. The average number of notifications per case was 2.1, taking into account sources of pathology and hematology. The percentage of unknown age in men and women was respectively 2.2% and 1.6%. The proportion of the cases (skin cancer excluded) with pathology confirmation was 97.9% in men and 98.6% in women.

### All cancer sites

**Figure 1:**
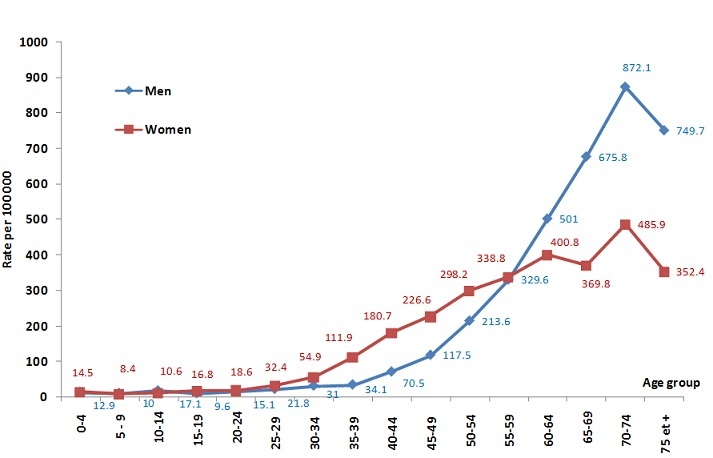
Age-Specific Incidence Rates of All Cancers in Greater Casablanca, 2005-2007

The incidence rates (crude, ASR and age-specific) of all types of cancer are displayed in Table 1. The age-standardized incidence rates (ASR) per 100,000 inhabitants for all sites were 120.7 in men and 115.9 in women. As shown in Figure 1, the age-specific incidence curve reveal that from age 30 to 55, incidence was higher in women while after age 60, incidence was higher in men, this being attributable in part to the young age of onset for breast and cervical cancer. As a result, cancer at young age in Morocco was more common in women, who represented 67% of the cases under 50. The median age of onset for cancer (any type) was 50.5 years in women and 56.6 years in men. The top 5 cancers in men were lung (ASR = 25.9), prostate (13.5), bladder (8.7), colon-rectum (8.1), and non-Hodgkin lymphoma (NHL) (7.2) (proportions: 22.1%, 10.5%, 7.0%, 7.2% and 6.6% of all cancers respectively). In women the top 5 cancers were breast (ASR = 36.4), cervical (15.0), thyroid (6.7), colon-rectum (5.8) and ovarian (5.3)(proportions: 34.3%, 13.3%, 6.5%, 5.0% and 4.7% of all cancers respectively).

**Table 1:** Numbers, crude and age-standardized incidence rates by cancer site, 2005-2007

Men	total	unk age	MV (%)	Crude rate	ASR world	Women	total	unk age	MV (%)	Crude rate	ASR world
**Oral cavity C00-08**	154	2	94.8	2.8	3.4	Oral cavity C00-08	120	0	95	2.1	2.4
**Pharynx C09-10, C12-14**	25	0	100	0.5	0.6	Pharynx C09-10, C12-14	9	0	100	0.2	0.2
**Nasopharynx C11**	209	3	99	3.8	4.2	Nasopharynx C11	67	1	98.9	1.2	1.2
**Esophagus C15**	55	1	100	1	1.2	Esophagus C15	53	2	95.1	0.9	1
**Stomach C16**	222	5	100	4.1	4.8	Stomach C16	147	3	99.2	2.6	2.7
**Colon, Rectum C18-20**	377	7	99.2	6.9	8.1	Colon, Rectum C18-20	306	6	98.4	5.4	5.8
**Anus C21**	35	1	100	0.6	0.8	Anus C21	27	0	100	0.5	0.6
**Liver C22**	31	1	88.9	0.6	0.7	Liver C22	21	0	96.7	0.4	0.6
**Gallbladder C23-24**	42	1	97.6	0.8	1	Gallbladder C23-24	96	3	91.5	1.7	1.9
**Pancreas C25**	49	0	90.5	0.9	1.1	Pancreas C25	45	1	75.5	0.8	0.9
**Nose, Sinus C30-31**	29	2	96.6	0.5	0.6	Nose, Sinus C30-31	15	0	100	0.2	0.2
**Larynx C32**	270	8	99.3	4.9	6.1	Larynx C32	31	3	100	0.6	0.5
**Lung C33-34**	1144	9	95.6	20.8	25.9	Lung C33-34	148	0	96.5	2.6	2.9
**Bone C40-41**	92	0	95.7	1.7	1.7	Bone C40-41	58	0	94.8	1	1.1
**Skin C44**	374	30	98.4	6.8	7.7	Skin C44	217	8	95.9	3.8	4.2
**Connective, Soft Tissue C47,49**	94	2	95.7	1.8	1.8	Connective, Soft Tissue C47, 49	88	3	94.3	1.5	1.6
**Breast C50**	38	1	100	0.7	0.8	Breast C50	2119	30	98.2	37.5	36.4
**Prostate C61**	548	15	99.1	10	13.5	Cervix uteri C53	816	8	98.5	14.4	15
**Other male genital C60, 62-63**	44	0	100	0.8	0.7	Uterus: corpus & NOS C54-55	154	2	99.4	2.7	3.1
**-**	-	-	-	-	-	Ovary C56	291	5	97.1	5.1	5.3
**-**	-	-	-	-	-	Other fem. genital C51-52, C57-58	107	4	100	1.9	2.2
**Kidney C64-66**	92	1	96.5	1.7	1.9	Kidney C64-66	66	0	95.6	1.2	1.5
**Bladder C67**	368	6	99.7	6.7	8.7	Bladder C67	52	0	99.4	0.9	1.1
**Brain, Nervous system C70-72**	164	3	100	3	3.3	Brain, Nervous system C70-72	158	1	98.1	2.8	3
**Thyroid gland C73**	68	1	97.6	1.2	1.4	Thyroid gland C73	401	2	98.2	7.1	6.7
**Hodgkin disease**	130	4	100	2.4	2.2	Hodgkin disease	130	1	100	2.3	2.1
**Non-Hodgkin lymphoma**	349	9	100	6.4	7.2	Non-Hodgkin lymphoma	251	10	100	4.5	4.7
**Leukemia**	140	0	100	2.6	2.7	Leukemia	107	1	100	1.9	2
**Other and unspecified** *	408	12	-	7.3	8.7	Other and unspecified*	272	6	-	4.8	5.2
**All sites**	5551	124	-	101.1	120.7	All sites	6372	100	-	112.7	115.9
**All sites but skin**	5177	94	97.9	94.3	113	All sites but skin	6155	92	98.6	108.9	111.7

* C17, C26, C37, C38, C48, C68-69, C74-75, C80, 973, 974, 975, 976, 995, 996. Unk: unknown; ASR: Age standardized rate; MV: Morphological verification

### Solid malignancies

**Figure 2:**
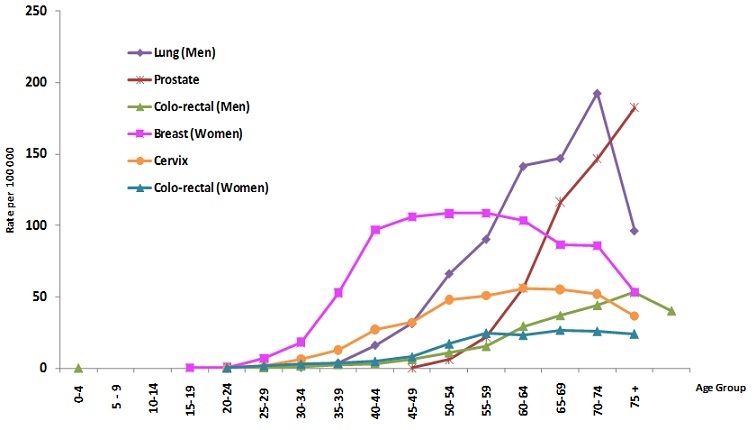
Age-Specific Incidence Rates of the Top 5 Cancers in Greater Casablanca 2005-2007

**Figure 3:**
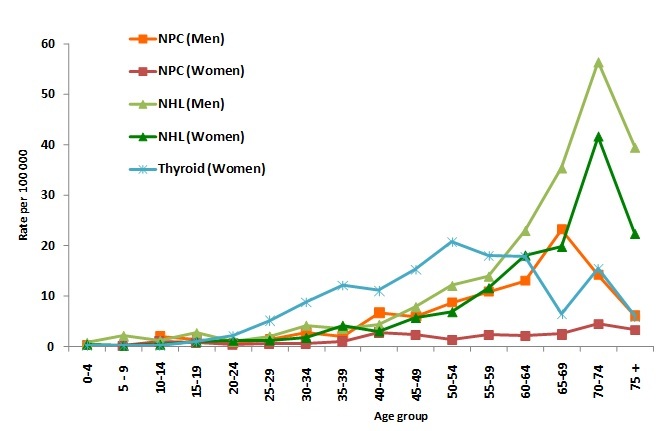
Age-Specific Incidence Rates of NPC, NHL and thyroid cancers in Greater Casablanca 2005-2007

For breast, cervical, colorectal and thyroid cancers a striking figure was that a large proportion of cases were diagnosed before age 50: 57% of breast cancer cases, 42% of cervical cancer cases, 28% of colorectal cancer cases (both sexes) and 60% of thyroid cancer cases (both sexes). For these 4 cancers the age specific incidence decreased after 60 or 70 years (Figure 2 for breast, cervical and colorectal cancer and Figure 3 for thyroid cancer). Age specific rates peaked at particularly young ages for female breast and thyroid cancer (age 50-60 and 50-54 respectively, Figure 2, Figure 3).

Lung cancer was much more frequent among men than among women (sex ratio M:F = 7.7) but the mean age of onset did not differ between men (59.5) and women (58.7). Sarcoma represented 0.1% of lung cancer in men and 1.4% in women. Similar features were observed for bladder cancer: higher incidence in men (sex ratio M:F = 6.9) and similar mean age of onset in both sexes (64.7 in men, 65.5 in women). Bladder squamous cell carcinomas were rare among men (1.4% of all bladder cancer) but represent a higher percentage among women (14.2%), however, the incidence of bladder squamous cell carcinomas was similar in both sexes, the difference in percentages was attributable to the very high sex ratio. Prostate cancer was the second most common cancer among men. We observed a marked 62% increase from 2004 (ASR = 9.6 95%CI= (7.9 - 11.3) to 2007 (ASR = 15.6 (13.4 - 17.8)). Breast and cervical cancers were respectively the first and second most common cancers in women. Over 4 years the ASR of breast cancer increased a little from 35.0 (32.3 - 37.9) in 2004 to 38.6 (35.8 - 41.4) in 2007, while the incidence of cervical cancer remained stable from 13.5 (11.7 - 15.3) in 2004 to 14.0 (12.2 - 15.8) in 2007. More than 75% of the breast cancers were of the ductal type. Squamous-cell carcinomas accounted for 77.3% of the cervical cancer, 8 % were adenocarcinomas, 2% were papillary carcinomas, 12% were of other histological types and 0.4% had unknown histology.

Nasopharyngeal cancer (NPC), which displays a higher incidence in Morocco than in Western countries [11] was the eighth cancer among men with an ASR of 4.2 and the 21th cancer among women with an ASR of 1.2 (sex ratio = 3.1). In both sexes the age-specific incidence displayed a little peak in the teens followed by a steady increase to reach a maximum at age of 65-69 for men and age of 70-74 for women (Figure 3).

Table 2 gives stage at diagnosis for breast, cervical and colorectal cancers; nasopharyngeal cancer was added since to our knowledge, population-based stages have never been published for a NPC endemic country out of Singapore [12]. For the 4 cancers the majority of the cases were diagnosed at regional stage. For prostate and lung cancer, respectively 56% and 42% of the medical records were missing stage data thus stage distributions are not presented.

**Table 2:** Stage at diagnosis for Breast, Cervix, Colorectal, and Nasopharyngeal cancer in Morocco, Saudi Arabia and the USA

Stage at diagnosis	Morocco	Saudi Arabia (2007) [16]	USA (2002-2008) [13]
**BREAST (women only)**			
Local	23%	14%	60%
Regional	52%	42%	33%
Distant	7%	13%	5%
Unstaged/missing	18%	32%	2%
**CERVIX**			
Local	18%		47%
Regional	42%	-	36%
Distant	4%		12%
Unstaged/missing	36%		6%
**COLORECTUM (both sex)**			
Local	9%	28%	39%
Regional	46%	35%	36%
Distant	13%	24%	20%
Unstaged/missing	32%	13%	5%
**NASOPHARYNGEAL (both sex)**		-	-
Local	9.5%		
Regional	58.5%		
Distant	8%		
Unstaged/missing	24%		

### Tumours of Haematopoietic and Lymphoid Tissues

Haematopoietic and Lymphoid malignancies are frequent in Morocco, together they represent 12.9% of all cancer types (non-melanoma skin cancers excluded) in men and 8.7% in women. The most frequent type was non-Hodgkin lymphoma (NHL) (ASR = 7.2 in men and 4.7 in women) followed by Hodgkin lymphoma (HL) (ASR = 2.2 in men and 2.1 in women) and by leukemia (ASR = 2.7 in men and 2.0 in women). We observed a 47% increase of NHL from ASR = 4.9 (4.1 - 5.7) (both sexes) in 2005 to ASR = 7.2 (6.2 - 8.2) in 2007. Out of all NHL patients, 38% were below age 50 and 8% below age 20. The disease was more frequent among men than among women. The sex ratio was 2.5 below age 20 and 1.5 above (p

HL affect young people, 75% of the patients were below age 50 and 20% below age 20. The mean age was 41.5 in men and 39.2 in women. The disease was equally frequent among men and women (ASR = 2.2 and 2.1 respectively). The histological type 2 nodular sclerosis was the more frequent affecting 50% of the patients. The ASR of leukemia was 2.7 in men and 2.0 in women; age specific incidence was bimodal with a little peak centred on age 5-9 and a highest one after 65.

### Cancer in children (0-14 years)

Childhood cancers represented 3% of all cancers with and ASR of 12.6, they were slightly more frequent in boys than girls (sex ratio 1.3). The leading childhood cancers, classified according to the International Classification of Childhood Cancer [13], were in both sexes: 1- CNS and miscellaneous intracranial and intra spinal neoplasms (18.2% of all tumours), 2- leukemia (10.9%), 3- retinoblastoma (10%), 4- bone tumours (10%), 5- renal tumours (8.1%), 6- NHL (7.3%), 7- HL (5.7%), 8- soft tissues and other extra osseous sarcomas (5%), 9- nasopharyngeal carcinoma (4.9%), and 10- Neuroblastoma and other peripheral nervous cell tumours (4.7%). ASRs for these cancers were respectively 2.3, 1.4, 1.4, 1.1, 1.1, 0.9, 0.7, 0.6, 0.5 and 0.5. The incidence of Burkitt Lymphoma was 0.3 (36% of all childhood NHL)

## Discussion

### Quality of data

This report presents the incidence of cancer in the Greater Casablanca district. Data quality and validity were reviewed by international experts and described as adequate [14]. Our case-finding approach has not changed since the first year of the registry (2004) so the degree of completeness of cancer registration is not expected to vary from one year to the other. The proportion of cases with histological confirmation (all sites except skin cancers) was 97.9% in men and 98.6% in women. These proportions are slightly above the ones observed in the west, for example in the 14 SEER registries (94.4% in men and 94.1% for women) [15] and the France-Herault registry (97.6% in both sexes) [16] and above those observed in Tunisia (92% in men and 95% in women for the central Tunisia registry) [17]. This suggests that our registry is rather too dependent on histopathology laboratories for case finding. As a result, incidence rates, especially those of cancers of the internal organs that are difficult to perform a biopsy on (liver, pancreas, gallbladder, etc.), may be under-estimated. This may also account, in part, for some of the apparent declines in incidence at old age, when diagnosis by histology is likely to be less common. In Morocco, under-diagnosis is relatively frequent among elderly since many, especially men, are reluctant to consult physicians or to pursue with full diagnosis once cancer is suspected. The local culture tends to the acceptation of disease and death as the natural consequence of old age.

Stage data were incomplete due to suboptimal documentation of stage in medical records. In our region, the cancer registry of the Saudi Kingdom reports stage data for 14 common cancers, however, missing stage proportions remain high (32% for breast, 46% for liver, 23% for lung), illustrating the difficulty to document stage even in a high-income country [18]. The International Agency for Research on Cancer (Lyon, France) conducted a large effort to retrieve and produce stage and survival date from 14 countries in Africa, Asia and Latin America (SurvCan study). The missing stage data for breast varied from 6.2% in Mumbai (India) to 52% in Hong-Kong (China) [12]. To improve stage notification in medical records, since 2008 our team has conducted numerous information/training meetings in health facilities of the Casablanca district, and it will continue to do so.

### Solid malignancies

Lung cancer was the first cancer in men; its incidence was close to that of the neighbour countries; it was half of the French one and a third of the USA one (Table 3). In Morocco in 2007, the prevalence of smoking was estimated to be 31.5% among men and 3.3% among women aged 15 and over [19] with nearly 41% of the population exposed to passive smoking [20]. Morocco has signed the WHO Framework Convention on Tobacco Control in 2004 and has a law that banned smoking in public places since the nineties, but it is not applied due the absence of a decree making the law enforceable. The Foundation Lalla Salma has developed an NGO-initiated program to fight tobacco entitled “Colleges, High Schools and Enterprises without Tobacco”. This program was officially launched on the World Day Without Tobacco in 2007.

**Table 3:** Comparison of ASRs with other countries for the 8 most common cancers (& NPC) in Morocco

	BREAST (women)	LUNG (men)	CERVIX (women)	PROSTATE (men)	COLORECTAL (men-women)	NHL (men)	BLADDER (men)	THYROID (women)	NPC (men)
Morocco (ASR) **(Greater Casablanca 2005-2007)**	36.4[34.8-38]	25.9[24.3-27.5]	15[13.9-16.1]	13.5[12.3-14.7]	8.1 – 5.8[7.2-9] – [5.1-6.5]	7.2[6.4-8]	8.7[7.8-9.6]	6.7[6-7.4]	4.2[3.6-4.8]
Algeria (ASR) [36]**(Oran 1996-2004)**	36.9	21.4	20.2	6.3	8.0 – 6.6	8.5	15.2	5.2	5.9
Tunisia (ASR) [15]**(North Tunisia 2004-2006)**	31.8	32.5	4.2	11.8	11.6 – 9.5	5.5	13.7	3.3	3.6
France (ASR) [37] **(Francim 2005)**	101.5	50.5	7.1	121.2	57.9 – 33.2	12.1	14.6	12.7	-
USA (ASR) [13]**(SEER 2004-2008)**	124	75.2	8.1	156	61.2 – 47.5	24	37.5	16.3	1

Breast cancer was the most common cancer in the population, both sexes considered; its incidence was 3 to 4 times lower than the one observed in Western countries (Table 3). Interestingly, age specific incidence in Moroccan women aged 65-69 was lower than incidence in women aged 40-44. This is most probably attributable to the evolution of reproductive habits: Moroccan women who are 65+ today are protected from breast cancer since they had large families, gave birth early and breastfed for long. A study conducted in 1987 on 5982 Moroccan women representative of the general population revealed that for women aged 45-49 (i.e. 65-69 in 2007), the mean number of children was 7.5, the mean age at first child delivery was 20.0 and the median duration of breast feeding was 15 months [21]. The same study conducted 14 years later revealed that these protective factors had decreased substantially: in women who were aged 40-44 in 2003, mean number of children was 4.5, mean age at first child delivery was 22.5 and median duration of breast feeding was 13.9 months [22]. Such an evolution in risk factor prevalence is expected to result in an increase of breast cancer incidence and indeed, over 4 years we observed a 10,3% increase from ASR = 35 (32.2 - 37.9) in 2004 to ASR = 38.6 (35.8 - 41.4) in 2007.

Cervical cancer was the 2nd cancer in women with an incidence comparable to the one of Algeria but 3 times higher than the one of Tunisia (Table 3). The low incidence of cervical cancer in Tunisia is well documented and has been attributed to monogamy and observance of legal age of marriage (17 years old in Tunisia) [23]. In Morocco and Algeria, polygamy is not forbidden and the legal age at marriage for girls (16 in Algeria, 18 in Morocco since 2003 and 15 before) is not enforced as much as in Tunisia [24].

In 2005-2007 there were no national cervical cancer or breast cancer screening programs in Morocco; this explains the relatively advanced stage at diagnosis for these two malignancies (Table 2) and the higher incidence of cervical cancer in Morocco when compared to Western countries. Early diagnosis programs are currently under implementation [25]; the Casablanca registry stage data will be a key in evaluating the impact of these programs. Interestingly, the stage at diagnosis of breast cancer was better in Moroccan than among Saudi citizens (Table 2); this may be attributed to differences in health system organizations. The media campaign targeting general cancer awareness, which ran in Morocco in 2006-07, may have also played a role. However, rates of localized breast cancer remain low in Morocco (23%) compared to the USA (60%, Table 2).

Colorectal cancer incidence was similar to the ones observed in Algeria and Tunisia, but 5 to 6 times lower than the ones observed in western countries. Colorectal cancer screening is not practiced in Morocco, nor in Algeria or Tunisia, however, the difference with Western countries may in majority be attributable to important differences in life-style. The Moroccan diet is rich in fruit and vegetables and very poor in processed food (until recently only unprocessed food items were available on the market). Furthermore, sedentary occupations are not as widespread in Morocco as in Western countries and 45% of the population is still rural [6].

For breast, cervical, colorectal and thyroid cancers a large proportion of cases were diagnosed before the age of 50. However when checked for age specific incidence below age 50 for these 4 cancers no difference was found compared to western population. For breast cancer for example, incidence in the age group 15-39 was 14.6, which is lower than the one of the USA (18.1) or France (23.6) [26].

The incidence of prostate cancer in Morocco (13.5) was higher than the one observed in Algeria (6.3) or Tunisia (11.8). This is mainly attributable to difference in screening practices; in Casablanca numerous private urologists encourage their patients to get screened using PSA tests. The 62% incidence increase observed in Casablanca between 2004 and 2007 confirm that the practice is recent. It correlates with the cancer awareness campaign conducted at the national level by the Foundation Lalla Salma since its creation in 2005. The incidence of prostate cancer remains 10 times lower than that reported in Western countries. This is attributable to major differences in screening, but the difference in diet may also play a role. North Africa is one of the regions of the world, together with Southern Asia, where prostate cancer incidence is the lowest [26]. It has been suggested that the Mediterranean diet may protect against prostate cancer [27]. Consumption of olive oil, tomato and allium vegetables, which are all very frequent in the Moroccan diet, have been suggested to be protective [28]. Our incidence of prostate cancer is also lower than those reported in sub-Saharan Africa; ASR of 39.6 and 26.0 have been reported by the Kampala registry (Uganda) [29] and the Harare registry (Zimbabwe) [30] respectively. Such differences suggest that North African men do not share the high susceptibility to prostate cancer observed in the population of sub-Saharan African origin [31, 32].

Bladder cancer was 7 times more frequent in Moroccan men than in Moroccan women, this may be attributable to the large difference in tobacco consumption between men and women. This difference in tobacco consumption may explain other features we observed in our data like an unusual peak of incidence observed for pancreatic cancer at age 55-65 in men but not in women (data not shown). It has been shown that smokers diagnosed with pancreatic cancer are on average 10 years younger than non-smokers [33].

The incidence of nasopharyngeal carcinoma in Morocco was similar to those of Tunisia and Algeria and higher than in Western countries (Table 3). The high incidence of nasopharyngeal carcinoma in Western North Africa has been well documented and is believed to result from the interactions of genetic, Epstein Barr virus infection and environmental factors, including diet at young age [11, 34, 35]. The age-specific incidence peak we observed in the teens is a characteristic of NPC in North Africa, this peak is also observed in Tunisia and Algeria but not in regions of high incidence (South China, South East Asia). In regions of low incidence, a peak is commonly seen in the age group 15-19 years [34]. In Morocco the peak is centred in the age group 10-14. The reasons for such a bi-modal age-specific incidence curve remain unclear [36]. The stage data we present are the first of its kind for North Africa. The SurvCan study provided NPC stage distribution in Singapore, and the situation was better than in Morocco: in 1993-1997, 18.2%, 36.3% and 3.5% of the cases were diagnosed at localized, regional and distant stage respectively, however, data were missing in 42% of the cases [12].

### Tumours of hematopoietic and lymphoid tissues

The incidence of NHL in Greater Casablanca (7.2 in men, 4.7 in women) was similar to that of Tunisia (5.5 in men, 3.8 in women) and Algeria (8.5 in men, 5.1 in women) but lower than observed in France (12.1 in men, 8.2 in women) or the USA (24 in men, 16.5 in women). The reason for such differences can be attributed in part to differences in diagnostic practices [37]. The increase in NHL cases we observed from 2005 to 2007 is most probably attributable to improved detection. In the recent past, slow growing lymphomas were not precisely diagnosed in Casablanca and were treated by general practitioners with corticoids. The important cancer awareness campaign initiated in 2006 at national level has resulted in rapidly changing practices. The sex ratio of 2.5 observed in the age group 0-19 has no clear explanation and may be attributable to small numbers as we had only 34 boys and 10 girls with NHL below age 20 in 2005-07. Surprisingly, we observed equal incidence of HL in both sex, while throughout the world the incidence in men is consistently higher than in women, with a sex ratio between 1.5 and 2 [37]. The reason for this difference is unclear to us.

### Childhood cancers

The incidence of childhood cancer in Morocco (12.6) was slightly under those of Tunisia (13.6) and Algeria (13.5) and under those of France (14.2) and the USA (14.1) [26]. The difference was mainly due to leukaemia for which ASR was only 1.4 in Morocco vs. 3.0 in Tunisia, 2.5 in Algeria, 4.0 in France or 5.6 in the USA. This low incidence is probably the result of under-diagnosis.

## Conclusion

The data of the Greater Casablanca cancer registry, despite some limitations in quality, have provided invaluable information on cancer incidence. They revealed the burden of cancers attributable to tobacco (lung cancer, upper aero-digestive tract and bladder cancer) in Morocco and triggered the implementation of tobacco control actions 40. Stage data and mortality should be considered for the next data collection.
